# Experimental Animal Models of Pancreatic Carcinogenesis for Prevention Studies and Their Relevance to Human Disease

**DOI:** 10.3390/cancers3010582

**Published:** 2011-02-09

**Authors:** Mami Takahashi, Mika Hori, Michihiro Mutoh, Keiji Wakabayashi, Hitoshi Nakagama

**Affiliations:** 1 Division of Cancer Development System, Carcinogenesis Research Group, National Cancer Center Research Institute, 1-1, Tsukiji 5-chome, Chuo-ku, Tokyo 104-0045, Japan; E-Mails: mihori@ncc.go.jp (M.H.); mimutoh@ncc.go.jp (M.M.); hnakagam@ncc.go.jp (H.N.); 2 Graduate School of Nutritional and Environmental Sciences, University of Shizuoka, Yada 52-1, Suruga-ku, Shizuoka 422-8526, Japan; E-Mail: gp1576@u-shizuoka-ken.ac.jp

**Keywords:** pancreatic cancer, hyperlipidemia, iNOS, hamster, BOP

## Abstract

Pancreatic cancer is difficult to cure, so its prevention is very important. For this purpose, animal model studies are necessary to develop effective methods. Injection of *N*-nitrosobis(2-oxopropyl)amine (BOP) into Syrian golden hamsters is known to induce pancreatic ductal adenocarcinomas, the histology of which is similar to human tumors. Moreover, K-*ras* activation by point mutations and *p16* inactivation by aberrant methylation of 5′ CpG islands or by homozygous deletions have been frequently observed in common in both the hamster and humans. Thus, this chemical carcinogenesis model has an advantage of histopathological and genetic similarity to human pancreatic cancer, and it is useful to study promotive and suppressive factors. Syrian golden hamsters are in a hyperlipidemic state even under normal dietary conditions, and a ligand of peroxizome proliferator-activated receptor gamma was found to improve the hyperlipidemia and suppress pancreatic carcinogenesis. Chronic inflammation is a known important risk factor, and selective inhibitors of inducible nitric oxide synthase and cyclooxygenase-2 also have protective effects against pancreatic cancer development. Anti-inflammatory and anti-hyperlipidemic agents can thus be considered candidate chemopreventive agents deserving more attention.

## 1. Introduction

In recent years, pancreatic cancer has increased to become the fifth leading cause of cancer mortality in Japan [1]. Since the five-year-survival rate is very low, elucidation of the mechanisms of pancreatic carcinogenesis and development of prevention methods are important high priority tasks. Factors affecting pancreatic cancer development have been studied using several *in vivo* animal models [2]. Use of *N*-nitrosobis(2-oxopropyl)amine (BOP) in the Syrian golden hamster is known to be unique for development of pancreatic ductal adenocarcinomas, the histology of which is similar to that in human cases [3-6]. In the hamster model, early lesions such as focal hypertrophy, hyperplasia, goblet cell metaplasia, atypical hyperplasia and *in situ* carcinoma sequentially develop in the common duct, pancreatic duct and ductules, but not in acinar cells [7]. Transplacental induction of pancreatic ductal cancer by 4-(methylnitrosamino)-1-(3-pyridyl)-1-butanone (NNK) and ethanol in Syrian golden hamster is also an interesting model to investigate a synergistic effect of cigarette smoking and alcohol drinking on fetuses [8]. In rats, the azaserine-induced pancreatic cancer model is well-known, but the lesions are acinar cell carcinomas [9]. A nitrosourea amino acid carcinogen, *N*-delta-(*N*-methyl-*N*-nitrosocarbamoyl)-L-ornithine (MNCO), has further been shown to cause pancreatic acinar cell carcinomas in rats [10] and ductal carcinomas in hamsters [11]. Different from in hamsters, BOP mainly induces thyroid gland tumors in rats [12,13] and lung and liver tumors in mice [14]. There is thus a species specificity in the types of pancreatic neoplasm induced in rodents [15-17]. The 7,12-dimethylbenzanthracene (DMBA)-induced pancreatic cancer model can also be employed as a chemical carcinogenesis model in rats [18,19] and mice [20]. In this case, direct implantation of the carcinogen into the head of the pancreas causes tubular complexes in acini and induces pancreatic neoplasms of ductal phenotype in which cytokeratin 19 is expressed [21] and K-*ras* gene mutations are present [22]. Recently, genetically engineered mouse (GEM) models of pancreatic exocrine cancer have been developed and used to elucidate mechanisms of pancreatic carcinogenesis, although the pathology is somewhat different from human cases [23]. Mouse models with pancreas-specific expression of mutant K-*ras* from the embryonic stage frequently develop acinar-to-ductal metaplasia and pancreatic intraductal neoplasms (PanINs), but few pancreatic cancers under normal conditions [24-26]. Additional alterations in tumor-suppressor genes, such as *p16* [27], *p53* [28], *dpc4* [29], and TGF-β receptor II [30], or pancreatitis [31] in the GEM models have been shown to cause quite high incidences of pancreatic cancers. On the other hand, conditional expression of mutant K-*ras* in the adult phase hardly induces PanINs and cancer if without pancreatitis [31]. Transgenic rats that express a mutated Ha- or K-*ras* oncogene regulated by the *Cre/lox* system have also been demonstrated to develop pancreatic ductal carcinomas upon injection of a *Cre*-carrying adenovirus into the pancreatic ducts and acini via the common bile duct [32,33]. In these rat models, mutant *Ras* is conditionally expressed in the pancreas of young adult rats and neoplastic lesions arise in pancreatic duct epithelium, intercalated ducts and centroacinar cells, but not acinar cells [32].

Here, we focus on the BOP-induced pancreatic cancer model in hamsters and discuss its utility for cancer prevention studies.

## 2. Genetic Alterations in Pancreatic Ductal Carcinomas of Humans and BOP-treated Hamsters

Pancreatic carcinogenesis is known to be a multi-step process involving multiple genetic alterations in humans [34-37] and similar genetic alterations have been found in hamsters [38,39]. Findings for genetic alterations in pancreatic ductal cancers in the two species are summarized in Table 1.

K-*ras* is quite frequently mutated in pancreatic ductal carcinomas in hamsters (70–95%) [40-42] as well as humans (75–100%) [43-45], resulting in activation of downstream signaling proteins such as elements in the Raf/MEK/MAPK and PI3K/Akt pathways. K-*ras* mutations are also observed in early lesions, such as atypical ductal hyperplasia in hamsters and humans [41,46]. The major K-*ras* mutation in BOP-induced pancreatic carcinomas in hamsters is predominantly a G to A transition in the second position of codon 12, while both G to A transitions and G to T transversions at the second position of codon 12 are frequently observed in human pancreatic cancers [43,44].

The *p16^INK4A^/CDKN2A* is known to be a tumor suppressor gene located at chromosome 9p that is inactivated in most pancreatic ductal carcinomas in humans (80–95%) by intragenic mutations (40%), homozygous deletions (40%) or hypermethylation of its promoter region (15%) [45,47,48]. The protein encoded by *p16* is an inhibitor of cyclin-dependent kinase and regulates the cell cycle by activation of RB proteins. Frequent alteration of *p16* (∼93%) has also been reported in BOP-induced pancreatic tumors in hamsters and the majority of changes involve aberrant methylation (47%) or homozygous deletion (37%) [49].

*DPC4/SMAD4* is a tumor suppressor gene located at chromosome 18q21.1 which encodes a protein associated with the TGF-β signaling pathway. *DPC4/SMAD4* is inactivated in 50% of pancreatic adenocarcinomas in humans by homozygous deletions (30%) or intragenic mutations in one allele coupled with loss of heterozygosity (LOH) (20%) [50]. On the other hand, *Dpc4/Smad4* alterations are rare in BOP-induced pancreatic tumors in hamsters (8%) [51].

*DCC* is a tumor suppressor gene located at chromosome 18q21.3, which encodes a protein with homology to cell adhesion receptors. Expression has been found to be lost in 50% of human pancreatic adenocarcinomas [52] and also in 50% of BOP-induced pancreatic tumors in hamsters [53]. In addition, DCC expression is reduced or lost in poorly differentiated or undifferentiated pancreatic cancer cell lines, whereas it is conserved in the more differentiated ones [52,37].

*p53* is the most frequently altered tumor suppressor gene in various cancers, its protein being a transcription factor which regulates cell cycle and apoptosis. *p53* is located at chromosome 17p and frequently inactivated by LOH and mutations in 40 to 75% of pancreatic adenocarcinomas in humans [34,45,54-56]. Overexpression of p53 protein can be detected in the nuclei of p53-mutated cells [54,55]. On the other hand, there is no evidence of p53 mutations in primary tumors in BOP-treated hamsters [57].

*FHIT* gene is a putative tumor suppressor gene located at chromosome 3p14, which is expressed in normal pancreatic ductular cells and is altered in pancreatic cancers [58]. Exogenous expression of *FHIT* in human pancreatic cancer cells causes cell cycle arrest and apoptosis [59] and loss of full length transcripts is frequent in primary pancreatic cancers of humans (62%) [58] and BOP-treated hamsters (73%) [60].

In addition to these gene alterations, increased protein expression, such as telomerase [61,62], midkine [63,64], cyclooxygenase-2 (COX-2) [65], metalloproteinase (MMP)-2, MMP-9 and membrane type 1-MMP [66,67] are shown in hamsters as in humans.

These findings indicate that multiple gene alterations and changes in protein expression observed in human pancreatic cancers are similarly involved in the BOP-induced hamster pancreatic ductal carcinogenesis model, underlining its utility for studying methods for pancreatic cancer prevention.

## 3. Modifying Factors in the Experimental Pancreatic Carcinogenesis Models

In addition to cigarette smoking, a well-known cause of pancreatic cancer, epidemiological studies have shown that chronic pancreatitis, obesity and diabetes mellitus are risk factors [68]. Using experimental animal models including mainly the BOP-induced pancreatic carcinogenesis model in hamsters, these and other possible promotive and suppressive factors in pancreatic carcinogenesis have been studied.

### 3.1. Obesity and Diabetes

Dietary fat has modifying effects on pancreatic carcinogenesis. It has been shown that a high-corn oil diet increased pancreatic ductal adenocarcinoma development in BOP-treated hamsters as compared with a low-corn oil diet [69]. Furthermore, a diet containing beef tallow has been shown to increase pancreatic cancer development compared with a diet containing corn oil [70]. Type and composition of fat are considered to be important. Fish oil rich in n-3 polyunsaturated fatty acids has been demonstrated to reduce pancreatic tumor incidences and hepatic metastasis in the BOP-treated hamster model [71]. Enhancing effects of high fat diet and suppressive influence of n-3 polyunsaturated fatty acid-rich fish oil on development of precancerous lesions, PanINs, in K-*ras* mutated GEM models have also been reported [72,73]. Obesity-mediated enhancement of PanIN lesion development is associated with increased inflammation, and abrogation of TNFR1 signaling blocks tumor promotion [72]. On the other hand, n-3 polyunsaturated fatty acids ameliorate inflammation through inactivation of the NF-κB signaling pathway and inhibit cell proliferation through induction of cell cycle arrest and apoptosis [73,74].

Streptozotocin is known to induce diabetes through damage to islet cells and its modifying effects on pancreatic carcinogenesis have been studied in the BOP-treated hamster model, though the results are somewhat controversial. It has been reported that administration of streptozotocin alone caused islet cell tumors (44%), pseudoductules (40%), and ductular adenomas (12%), while simultaneous treatment with streptozotocin (single i.v. injection, 30 mg/kg body weight) and BOP (single s.c. injection, 10 mg/kg body weight) resulted in a significantly higher incidence of ductular carcinomas than induced by BOP alone [75]. On the other hand, pretreatment with streptozotocin at a diabetogenic dose (50 mg/kg body weight, three-times i.p. injection) prevented pancreatic cancer development when BOP was subsequently administered [76]. These inhibitory effects of pretreatment were dependent on the severity of the diabetes and could be blocked with nicotinamide [77]. These findings indicate that streptozotocin has a tumorigenic activity at relatively low dose, but when administered before BOP treatment, streptozotocin-induced diabetes/loss of insulin production could prevent BOP-induced pancreatic cancer development through killing islet cells. However, enhancing effects of diabetes and insulin-resistance observed in obesity on growth of transplantable pancreatic cancer cells are nevertheless convincing [78-80].

### 3.2. Pancreatitis

Cerulein is an analogue peptide of cholecystokinin, and its chronic intraperitorial injection causes pancreatic hypertrophy, characterized by increased pancreatic weight, increased amylase content and acinar cell hyperplasia. Moreover, cerulein augments the carcinogenicity of *N*-nitrobis(2-hydroxypropyl)amine (BHP) in the hamster pancreas [81]. It is also reported that chronic pancreatitis caused by cerulein induces development of pancreatic ductal adenocarcinomas in GEM mice expressing K-*ras*^G12V^ in acinar/centroacinar cells [31]. On the other hand, pancreatitis caused by common duct ligation before BOP injection decreased carcinoma development, while repeated induction of pancreatitis by common duct ligation after BOP administration resulted in enhanced development of carcinomas, with reference to both number and size [82].

Heavy alcohol drinking and cigarette smoking are major causes of pancreatitis in humans [83]. Epidemiological studies have shown that smoking and chronic pancreatitis are risk factors, whereas alcohol consumption itself has no direct relation [83,84]. However, in a transplacental induction model of pancreatic ductal cancer featuring NNK and EtOH treatment in the Syrian golden hamster, EtOH alone caused pancreatitis and hyperplasia, while NNK alone did not induce either [8], indicating a strong enhancing effect of pancreatitis on pancreatic carcinogenesis. It has also been reported that EtOH and nicotine promote pancreatic carcinogenesis in the DMBA-implanted mouse model [85,86].

In addition, repeated induction of pancreatitis with choline-deficient diet combined with DL-ethionine and L-methionine after initiation with BOP has been demonstrated to cause rapid production of pancreatic carcinomas in hamsters [87].

### 3.3. Others

There is limited evidence suggesting that red meat is a cause of pancreatic cancer [88,89]. In addition to total intake, the method of meat preparation is also important. Grilled red meat is a risk factor [90]. Effects of mutagenic heterocyclic amines (HCA) formed during cooking of meat on pancreatic carcinogenesis were studied in the BOP-treated hamster model. Among HCAs, 3-amino-1,4-dimethyl-5*H*-pyrido[4,3-*b*]indole (Trp-P-1) and 2-amino-3,4,8-trimethylimidazo[4,5-*f*]quinoxaline (4,8-DiMeIQx) caused increase in pancreatic carcinoma development in BOP-treated hamsters [91]. Dietary intake of DiMeIQx has also been shown to be associated with pancreatic cancer risk in man [92].

High intake of fruits, vitamin C and vitamin E are suggested to protect against pancreatic cancer [88,93,94] and both vitamins have been found to exert protective effects on BOP-induced pancreatic cancer development in hamsters [95].

## 4. Cancer Prevention Targets for Humans and Evaluation in Experimental Pancreatic Carcinogenesis Models

From the etiology of pancreatic cancer, possible methods for prevention are: (1) avoiding carcinogenic *N*-nitrosoamine exposure such as cigarette smoke; (2) body weight control by diets and physical activity; (3) use of anti-hyperlipidemic and/or anti-diabetic agents; (4) use of antiinflammatory agents.

Epidemiological studies have suggested that several agents having anti-hyperlipidemic, antidiabetic or anti-inflammatory activities may have chemopreventive potential against pancreatic cancer [96]. Statins are cholesterol-lowering agents and also inhibit membrane-binding of the Ras protein, and are reported to reduce pancreatic cancer cell invasion and metastasis [97]. A casecontrol study of half a million veterans demonstrated a significant reduction of pancreatic cancer risk in statin users (adjusted OR = 0.37) [98], while meta-analysis of 12 studies showed no evidence of association between statin use and pancreatic cancer risk (RR = 0.88) [99]. Aspirin is a most frequently used nonsteroidal anti-inflammatory drug (NSAID) and has been reported to reduce cancer risk in several organs such as in the colon [100]. In the pancreas, epidemiological data on aspirin use are controversial [101,102]. A cohort study of post-menopausal women has shown that current use of aspirin is associated with a reduced risk of pancreatic cancer (adjusted RR = 0.57) [103], whereas another cohort study of nurses demonstrated that more than 20 years of regular aspirin use is associated with increased risk (RR = 1.58) [104]. Metformin, an anti-diabetic drug, activates AMP-activated protein kinase (AMPK) and inhibits pancreatic cancer growth [105,106]. A hospitalbased case-control study has shown that metformin use is associated with reduced risk (OR = 0.38), while insulin or insulin secretagogue use is associated with increased risk (OR = 1.78) of pancreatic cancer in diabetic patients [107]. However, there is still no report of cohort study or randomizedcontrol trial on metformin use. Since incidence of pancreatic cancer is relatively low compared with colon, breast and prostate cancers, prospective studies need quite a large population. In addition, randomized control studies are difficult, because the diseases such as hyperlipidemia and diabetes should be properly cared for. Therefore, evidences provided by preclinical studies including *in vivo* carcinogenesis studies using animal models are considered to be very important to evaluate the chemopreventive efficacy and mechanisms of these agents. Factors related to insulin resistance and inflammation are candidate targets for pancreatic cancer prevention. Table 2 shows chemopreventive agents evaluated in BOP-induced pancreatic carcinogenesis.

### 4.1. Anti-Hyperlipidemic/Diabetic Agents

It has been reported that high cholesterol intake is associated with an increased risk of pancreatic cancer [108]. Smoking is associated with metabolic syndrome, and nicotine elevates serum triglyceride levels [109,110]. Obesity and diabetes are also closely associated with hyperlipidemia and hyperinsulinemia [111,112]. Interestingly, Syrian golden hamsters are in a hyperlipidemic state even under normal diet conditions, and pioglitazone, a ligand of peroxizome proliferator-activated receptor (PPAR) γ, has demonstrated to improve hyperlipidemia and suppress development of ductal adenocarcinomas in BOP-treated hamsters; the ductal adenocarcinoma incidences in the BOP + 800 ppm pioglitazone group and the BOP alone group were 38% *vs.* 80% (*P* < 0.01) and the multiplicities were 0.55 ± 0.15 *vs.* 1.37 ± 0.22 (*P* < 0.01), respectively [113]. In addition, the incidences of bile duct tumors in BOP-treated hamsters were clearly suppressed by pioglitazone [113]. Metformin, an activator of AMPK, has also been shown to decrease serum insulin levels and suppress development of hyperplastic, dysplastic and malignant ductal lesions in the pancreas of BOP-treated hamsters on a high fat diet condition [114]. Pioglitazone and metformin are both anti-diabetic drugs which improve insulin resistance [115].

### 4.2. Anti-inflammatory Agents

Expression of COX-2 is up-regulated in PanIN and adenocarcinomas in humans and BOP-treated hamsters [64,116-118] and inhibition of prostanoid synthesis by NSAIDs, such as indomethacin and phenylbutazone, has been shown to reduce the development of precancerous lesions (atypical hyperplasia) and adenocarcinoma in the hamster model [119,120]. Whereas suppressive effects of aspirin were not significant, nitric oxide (NO)-donating aspirin, NO-ASA, has potent activity to prevent pancreatic cancer, especially arresting the transition from PanIN2 to PanIN3 and carcinoma, in BOP-treated hamsters [121]. It has also been reported that another COX-inhibitor, ibuprofen, reduces pancreatic cancer development in the hamster transplacental model with NNK + EtOH [122]. In GEM models, aspirin treatment has been shown to delay progression of PanINs in *LsL- Kras^G12D^; Pdx1-Cre* mice and to partially inhibit development of invasive cancers in *LsL-Kras^G12D^; LsL-Trp53^R172H^; Pdx1-Cre* mice [123]. Furthermore, a selective COX-2 inhibitor, nimesulide, has been demonstrated to suppress development of precancerous lesions (atypical hyperplasia) and adenocarcinoma in BOP-treated hamsters [118]. In addition, inhibition of COX-2 by nimesulide delayed the appearance of PanIN-2 and PanIN-3 lesions in a conditional K*ras^G12D^* mouse model, indicating the importance of prostaglandin synthesis by COX-2 in the early stage of pancreatic carcinogenesis [124]. In addition to COX-2, 5-LOX is also up-regulated in the ductal cells of PanIN and adenocarcinomas in humans, BOP-treated hamsters and Elastase-K*ras* mice [125,126]. Receptors of the downstream 5-LOX metabolite, leukotriene B_4_, have been reported to be expressed in human pancreatic cancer tissues [125] and combination of COX-2-inhibition by Celebrex and 5-LOXinhibition by Zyflo has shown to significantly decrease liver metastasis by pancreatic cancers in BOPtreated hamsters [127]. MK886, an inhibitor of 5-LOX activating protein FLAP, also reduced pancreatic cancer development in the hamster transplacental model with NNK + EtOH [122].

Increased expression of iNOS is also observed in pancreatic adenocarcinomas in humans and hamsters [128-131], perhaps involving K-*ras* activation [132]. Inhibition of iNOS by a selective iNOS inhibitor ONO-1714 suppressed development of precancerous lesions (atypical hyperplasia) and invasive adenocarcinomas in BOP-treated hamsters [131].

### 4.3. Others

Expression of MMP-2 is increased in precancerous lesions and adenocarcinomas, and proMMP-2 is highly activated in pancreatic carcinomas in humans and hamsters [133,66]. Inhibition of proMMP-2 activation by the MMP inhibitor OPB-3206 has been demonstrated to suppress pancreatic cancer development in BOP-treated hamsters under a rapid production protocol [66]. Another MMP inhibitor, RO 28-2653, has been reported to inhibit liver metastasis in the BOP-induced pancreatic carcinogenesis model, directly indicating roles for MMP-2 in cancer progression [134].

Protochatechuic acid, green tea extracts (GTE) and butylated hydroxyanisole (BHA) are antioxidative agents which have demonstrated inhibitory effects on pancreatic cancer development during the post-initiation stage of the BOP-initiated hamster model [135-137]. Sarcophytol A, which is known to be an anti-tumor promoter, and methionine, which is an essential amino acid and associated with anti-oxidation, have also been shown to suppress pancreatic carcinogenesis in the BOP-treated hamster model [138,139].

Phenethyl isothiocyanate (PEITC), a natural constituent of cruciferous vegetables, has been demonstrated to be a potent chemopreventive agent in the initiation phase of pancreatic carcinogenesis in hamsters initiated with BOP [140,141], while not affecting the post-initiation phase [142]. Synthetic analogues of PEITC, such as 3-phenylpropyl isothiocyanate (PPITC), 4-phenylbutyl isothicyanate (PBITC) and benzyl isothicianate (BITC), and sulforaphane, Aloe arborescens and oltipraz have also been shown to suppress the initiation phase of BOP-induced pancreatic carcinogenesis through inhibition of activating (phase I) enzymes or activation of detoxifying (phase II) enzymes related to metabolism of BOP [143-147].

Nicotine-derived nitrosamine NNK stimulates release of noradrenaline/adrenaline by binding to alpha7 nicotinic acetylcholine receptors and activates beta-adrenergic receptors, resulting in proliferation of human pancreatic epithelial cells through cAMP-dependent signaling [148,149]. A beta-blocker propranolol has been shown to suppress the development of pancreatic cancer induced in the hamster transplacental model with NNK + EtOH [150].

Angiotensin-I-converting enzyme (ACE) and angiotensin II type 1 receptor are upregulated in human pancreatic cancer tissues and co-localized with vascular endothelial growth factor (VEGF) in malignant ducts and in stromal cells [151]. The ACE inhibitor enalapril has been demonstrated to delay progression of PanINs in *LsL- Kras^G12D^; Pdx1-Cre* mice and to partially inhibit development of invasive cancer in *LsL-Kras^G12D^; LsL-Trp53^R172H^; Pdx1-Cre* mice [123].

An epidermal growth factor receptor inhibitor, gefitinib, has been demonstrated to suppress development of PanINs and cancer in *LsL-Kras^G12D^; p48-Cre* mice [152]. Furthermore, a src kinase inhibitor, dasatinib, has been shown to suppress metastasis in *LsL-Kras^G12D^; LsL-Trp53^R172H^; Pdx1-Cre; Z/EGFP* mice, although there are no effects on proliferation and no survival advantage [153]. In addition, synthetic oleanane triterpenoids CDDO-methyl ester or CDDO-ethyl amide, the rexinoid LG100268, or the combination have been shown to increase survival in *LsL-Kras^G12D^; LsL-Trp53^R172H^; Pdx1-Cre* mice [154].

## 5. Conclusions

As shown above, the BOP-induced pancreatic carcinogenesis model in Syrian golden hamsters has genotypic and phenotypic similarities to the human case, and is a useful animal model for investigation of cancer prevention, even though the mechanistic analyses are a little difficult due to its limited genetic information. In this model, both precancerous lesions and advanced ductal carcimomas are assessable, and most of the BOP-treated hamsters develop pancreatic ductal carcinomas within six months. On the other hand, DMBA-induced pancreatic carcinogenesis models in rats and mice are considered to be not suitable for prevention studies, from the viewpoints of pathological origin of cancers and technical difficulty with neoplastic lesions developing only where carcinogen is implanted. GEM models are powerful for verifying the oncogenic mechanisms, but the process of carcinogenesis is pathologically different from the vast majority of human cases. Recently, several chemoprevention studies using GEM models have been reported [73,123,124,126, 152-154], mainly of two types. One focuses on suppression of PanIN development in *LsL- Kras^G12D^; Pdx1-Cre* mice or *LsL- Kras^G12D^; p48-Cre* mice. In this system, incidences of pancreatic cancer are low (∼20% at one year) [155], and therefore, it is difficult to obtain statistically significant results for cancer development. The PanIN lesions in GEM mice have similar phenotypes to humans, such as COX-2 [124] and LOX-5 [126] expression, but the pathological process of development of early lesions is quite different from human cases. Thus, the usefulness of this model may be limited regarding early detection and prevention of human pancreatic cancer. In suppression studies on cancer development or prolonged survival with *LsL-Kras^G12D^; LsL-Trp53^R172H^; Pdx1-Cre* mice, the GEM animals mimic the genetics of human pancreatic cancer and quickly develop pancreatic ductal carcinomas. This model may be more suitable for therapeutic studies than for prevention.

In humans, a number of epidemiological studies have suggested reduced pancreatic cancer risk with use of anti-hyperlipidemic/diabetic or anti-inflammatory agents. However, this is difficult to prove in randomized-control studies, because of the relatively low incidence of pancreatic cancer in humans and the absence of early biomarkers to predict pancreatic cancer. Thus, *in vivo* carcinogenesis studies using animal models are important to support the epidemiological findings and provide direct evidence. Some anti-hyperlipidemic and anti-inflammatory agents have indeed been shown to exert suppressive effects on pancreatic carcinogenesis in animal models including that with BOP-initiation in the hamster, indicating that factors related to hyperlipidemia, insulin resistance and inflammation are candidate targets for pancreatic cancer prevention.

## Figures and Tables

**Table 1.** Gene alterations in pancreatic cancers in humans and hamsters [34-60].

**Gene**	**Alterations**	**Frequency in (%)**	

		**Human**	**Hamster (BOP-treated)**
*K-ras*	Mutation	75–100	70–95
*p16^INK4A^/CDKN2A*	CpG methylation/Deletion/Mutation	80–95	93
*DPC4/SMAD4*	Deletion / Mutation + LOH	50	8
*DCC*	Deletion	50	53
*P53*	Mutation + LOH	40–75	0
*FHIT*	Aberrant transcripts	62	73

LOH: loss of heterozygosity

**Table 2.** Chemopreventive agents of *N*-nitrosobis(2-oxopropyl)amine (BOP)-induced pancreatic carcinogenesis in hamsters.

**Compounds**	**Mechanistic categories**	**Ref.**
Anti-hyperlipidemic/diabetic agents		
Pioglitazone	PPARγ ligand	[113]
Metformin	AMPK activator	[114]
Anti-inflammatory agents		
Indomethacin	NSAID	[119]
Phenylbutazone	NSAID	[119]
NO-ASA	NO-NSAID	[121]
Nimesulide	COX-2 inhibitor	[118]
Celecoxib/Zileuton	COX-2/5-LOX inhibitors	[127]
ONO-1714	iNOS inhibitor	[131]
Others		
OPB-3206	MMP-2 inhibitor	[66]
Protochatechuic acid	Antioxidant	[135]
GTE	Antioxidant	[136]
BHA	Antioxidant	[137]
Sarcophytol A	Anti-tumor promoter	[138]
Methionine	Essential amino acid	[139]
PEITC	Cytochrome P450 suppressor	[140]
PPITC	Cytochrome P450 suppressor	[143]
PBITC	Cytochrome P450 suppressor	[144]
BITC	Cytochrome P450 suppressor	[145]
Sulforaphane	Anti-oxidative enzyme inducer	[145]
Aloe arborescens	Detoxyfiying enzyme inducer	[146]
Oltipraz	Nrf2 activator	[147]
